# Effect of intravitreal ranibizumab on the ocular circulation of the untreated fellow eye

**DOI:** 10.1007/s00417-017-3692-z

**Published:** 2017-06-28

**Authors:** Masahiko Sugimoto, Takayasu Nunome, Rie Sakamoto, Maki Kobayashi, Mineo Kondo

**Affiliations:** Department of Ophthalmology, Mie University Graduate School of Medicine, 2-175 Edobashi, Tsu, 514-8507 Japan

**Keywords:** Laser speckle flowgraphy, Ocular blood circulation, Macular edema, Ranibizumab, Systemic circulation

## Abstract

**Purpose:**

To evaluate the effects of unilateral intravitreal ranibizumab (IVR) on the ocular circulation of the fellow eyes.

**Methods:**

Fifteen eyes of 15 patients with macular edema (average age 69.6 ± 11.8 years) were studied. Eleven eyes had diabetic macular edema (DME) and four eyes had macular edema associated with a branch retinal vein occlusion. Each eye received 0.5 mg of IVR. The blood circulation on the optic nerve head of the treated and untreated eyes were determined by laser speckle flowgraphy (LSFG, Softcare Co., Ltd) before, 1 day, and 1 week after the IVR. The mean blur rate (MBR) and the relative changes of the MBRs determined as dMBR(%) = 100−(MBR before/MB after) × 100) were evaluated. The central macular thickness (CMT) and the rate of reduction in the thickness (dCMT = 100−(CMT before/CMT after) × 100) were also evaluated.

**Results:**

The mean dMBR was significantly higher in the treated eyes than the untreated eyes at 1 day (−16.4 ± 17.0% vs 2.31 ± 19.3%) and at 1 week (−12.0 ± 14.6% vs 4.50 ± 25.9%) after the IVR (*P* = 0.02, paired *t* tests).

**Conclusion:**

These findings indicate that if ranibizumab enters the systemic circulation, the concentration is not high enough to affect the ocular circulation of the fellow eyes.

## Introduction

Anti-vascular endothelial growth factor (anti-VEGF) agents have become the first-line therapy for various eye diseases including age-related macular degeneration (AMD) [1, 2], retinal vein occlusion related macular edema (RVO-ME) [3], and diabetic macular edema (DME) [4]. VEGF plays an important role in vascular angiogenesis and vascular homeostasis in eyes after ischemia-reperfusion due to an infarction. These properties indicate that intravitreal injections of anti-VEGF agents may be able to resolve problems of ocular neovascularization but may also cause thrombosis and re-occlusion of new vessels [5]. Fung et al. conducted an internet survey of 70 ophthalmological centers that had performed 7,113 injections of anti-VEGF agents in 5,228 patients. They concluded that intravitreal injections of bevacizumab (IVB; Avastin®, Roche, Basal, Schweiz, IVB) caused systemic complications in about 1 in 3,000 patients [6]. Another study reported on the relationship between anti-VEGF agents treatment and cardiac complications [7].

In addition, recently, large scale data (from 57,919 patients) mentioned high incidence of systemic adverse event after anti-VEGF treatment [8]. On the other hand, a meta-analysis of 21 randomized cohort studies [9] and comparison of ten RCT trials [10] reported that the frequency of these complications is not so high. Thus, there remain a controversy about systemic circulation of anti VEGF agents. It is still unclear whether anti-VEGF agents can cause systemic side-effects. And to have systemic affects, the anti-VEGF agents must pass from the vitreous into the systemic circulation at high enough concentrations to be effective.

The characteristics of the ocular blood flow can be determined by scanning laser ophthalmoscopy, color Doppler imaging (CDI), laser speckle flowgraphy (LSFG), and some other methods. LSFG can determine the blood flow velocity in ocular tissues including that in the retinal and choroidal vessels with a good reproducibility, using a bundled software [11–13]. Its usefulness in monitoring the same site during a disease process noninvasively has been established, and it is also known to correlate with improvement of visual function [14].

Thus, the purpose of this study was to determine whether a unilateral intravitreal injection of ranibizumab (IVR; Lucentis®, Novartis Phamaceuticals, Cambridge, MA, USA), one of the major anti-VEGF agents, will affect the ocular circulation of the fellow eye. To accomplish this, ranibizumab was injected intravitreally into one eye, and the ocular circulation in both the injected and the fellow eye was determined by LSFG.

## Material and methods

### Participants

This study was conducted at the Mie University Hospital from November 2014 to February 2015 and included 15 eyes of 15 patients; 11 eyes with DME and four eyes with macular edema associated with a retinal vein occlusion (RVO). All of the patients agreed to participate in this study, and the informed consent form was approved by the Institutional Review Board of the Mie University Hospital (No. 2842). The study was registered International Clinical Trial Registry Platform (UMIN 000021643).

### Inclusion and exclusion criteria

The inclusion criteria were: unilateral macular edema (ME), age ≥ 20 years, and best-corrected visual acuity (BCVA) ≥20/320. The diagnosis of ME was made by the clinical findings and spectral-domain optical coherence tomographic (SD-OCT) findings. The exclusion criteria were: ocular surgery, macular laser photocoagulation, and intravitreal or subtenon injections of any drugs within 2 months of the IVR injection. In addition, eyes with ocular inflammation, drusen, severe proliferative diabetic retinopathy, retinal hemorrhage which involved the intra- or subfoveal spaces, an epiretinal membrane, prior pars plana vitrectomy, glaucoma, and media opacities that significantly affected the BCVA were excluded. Patients with uncontrolled systemic medical conditions or history of thromboembolic events or ischemic diseases were also excluded.

### Intravitreal ranibizumab (IVR) injection

Each eye received a single injection of 0.5 mg of intravitreal ranibizumab (IVR) under local subconjunctival anesthesia. Each patient received ranibizumab intravitreally through a 30-gauge needle that was inserted 4 mm posterior to the corneal limbus under sterile conditions. All patients received topical levofloxacin hydrate (1.5% Cravit ophthalmic solution®, Santen Pharmaceutical Co., Ltd., Osaka, Japan) after the injection.

### Ophthalmic evaluations

Each examination was performed at the baseline, and 1 day and 1 week after the IVR injection. The intraocular pressure (IOP) was measured with a noncontact tonometer (NT-530, NIDEK, Gamagori, Aichi), and the degree of ME was determined from the images recorded by a Heidelberg Spectralis OCT instrument (Heidelberg Engineering Inc., Heidelberg, Germany). For qualitative and quantitative analyses of the OCT images, the fast macula protocol was used to obtain images with an automatic real-time calculation of the mean value of 9 which acquires 25 horizontal lines consisting of 1024 A-scans/line. The central macular thickness (CMT) was defined as the thickness between the internal limiting membrane and the retinal pigment epithelium at the fovea, and the value was automatically calculated for the central subfields of the macular thickness map using the bundled software.

### Measurement of ocular blood flow by LSFG (Fig. 1)

The LSFG instrument (Softcare Co., Ltd., Fukuoka, Japan) was used to measure the blood flow on the optic nerve head based on the laser speckle phenomenon [15]. This instrument consisted of a fundus camera equipped with an 830 nm diode laser. The laser light was used to measure an area of 3.8 × 3 mm with an estimated tissue penetration of 0.5 to 1.0 mm [11]. The scattered laser light from the target area was recorded with a camera with a 700 × 480-pixel resolution. Each image was transferred to a digital file and analyzed using the embedded software (LSFG Analyzer version 3.0.47.0, Softcare Co., Ltd., Fukuoka, Japan).

**Fig. 1:**
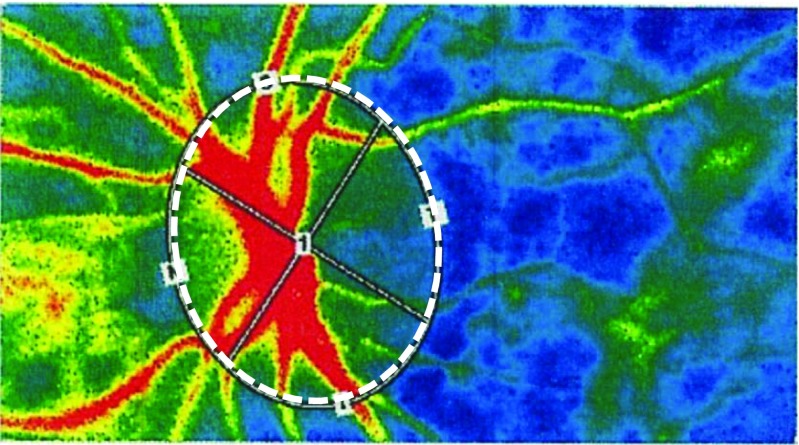
Composite LSFG color map of the MBR of a healthy eye. The *red color* indicates high MBR, the *blue color* indicates low MBR. *White-dot circle* shows the area around optic nerve head where the MBR was measured by LSFG. *LSFG*, laser speckle flowgraphy; *MBR*, mean blur rate

The mean blur rate (MBR), a quantitative index of the relative blood flow velocity, was used for the analyses [11, 16–18]. The MBR is reported as a good index for the actual blood flow , with a high correlation. [19, 20]. Its reproducibility, especially on the ONH, is reported to be good [21]. So, here we used MBR as indicator. The MBR was measured around the optic nerve head (ONH) for both the treated and untreated eye before, and 1 day and 1 week after the IVR injection. To compare the MBR change between different eyes, it is not appropriate to compare MBR value itself because the basic status of each eye is not necessarily the same. So here, to evaluate the relative changes in the MBR, we used dMBR expressed as a rate of change from the baseline values; dMBR was defined as follows:

$$ \mathrm{dMBR}\ \left(\%\right)=100-\left(\mathrm{MBR}\ \mathrm{after}\ \mathrm{IVR}/\mathrm{MBR}\ \mathrm{before}\ \mathrm{IVR}\times 100\right). $$

### Systemic hemodynamics

The systolic blood pressure (SBP) and diastolic blood pressure (DBP) were measured at the upper arm with a sphygmomanometer. Examinations were performed in the sitting position after a rest of 5 to 10 min. The mean arterial pressure (MAP) was calculated as MAP = DBP + 1/3 (SBP−DBP), and the ocular perfusion pressure (OPP) as OPP = 2/3 MAP−IOP.

### Statistical analyses

The data are presented as the means ± standard deviations (SDs). Inter-group comparisons were done by two-way analysis of variance (ANOVA), and the Tukey–Kramer test was used as a post hoc test. Paired *t* tests were used to compare the groups.

## Results

**Fig. 2:**
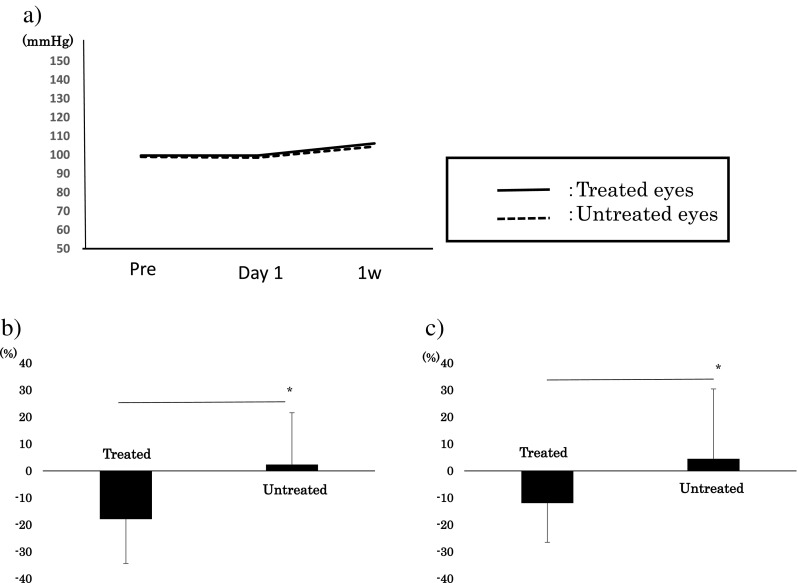
Comparisons of the changes in the mean blur rates in the IVR-treated and untreated eyes. **a** No significant difference in the OPP is present in the treated and untreated eye. **b** There was a significant difference between the relative MBR (ratio of MBR after/before IVR) of the treated eyes and untreated eyes at 1 day after the IVR (*P* = 0.01, paired *t* test, *: *P* < 0.05). **c** There was also a significant difference in the ratio of the relative MBR between the treated eyes and untreated eyes at 1 week after treatment (*P* = 0.03, paired *t* test *: *P* < 0.05). *IVR*, intravitreal ranibizumab; *MBR*, mean blur rate; *OPP*, ocular perfusion pressure

The demographics of the patients are shown in Table 1. The differences in the age, sex distribution, IOP, BP, pulse, and OPP between the DME and RVO-ME groups at the baseline were not significant. The differences in the baseline IOP and OPP between the treated and untreated eyes were also not significant. The OPPs before, and 1 day and 1 week after treatment were not significantly different (Fig. 2a, repeated ANOVA).

**Table 1:** Patient background

	Total patients (*n* = 15)	DME (*n* = 11)	BRVO-ME (*n* = 4)
Age (years)	69.6 ± 11.8	67.7 ± 13.4	73.0 ± 9.1
Gender (M:F)	7:8	4:7	3:1
Baseline IOP (mmHg)	Treated:13.0 ± 3.0 Untreated:13.7 ± 2.2	Treated:13.5 ± 3.3 Untreated:13.9 ± 2.4	Treated:11.6 ± 1.4 Untreated:13.7 ± 2.2
Baseline BP (systolic mmHg)	144.8 ± 14.0	146.1 ± 15.5	141.3 ± 9.3
Baseline BP (diastolic mmHg)	72.4 ± 13.1	70.2 ± 13.1	72.4 ± 13.1
Baseline pulse (bpm)	83.3 ± 10.6	84.5 ± 11.3	79.8 ± 8.8
Baseline OPP (mmHg)	Treated:99.6 ± 12.2 Untreated:99.0 ± 12.3	Treated:100.7 ± 14.0 Untreated:100.4 ± 13.9	Treated:96.6 ± 5.0 Untreated:95.1 ± 5.6

*BP* blood pressure, *BRVO* branch retinal vein occlusion, *DME* diabetic macular edema, *IOP* intraocular pressure, *ME* macular edema, *OPP* ocular perfusion pressure

The mean baseline CMT was 440.9 ± 160.0 μm, and it decreased significantly to 405.7 ± 149.9 μm at 1 day after the IVR, and to 330.9 ± 79.2 μm at 1 week after the IVR (*P* < 0.05 for both, repeated ANOVA). For the DME group, the mean baseline CMT was 389.8 ± 124.4 μm, and it decreased to 384.6 ± 142.6 μm at 1 day after the IVR. In addition, it significantly decreased to 305.0 ± 63.9 μm at 1 week after the IVR (*P* < 0.05, repeated ANOVA). The dCMT between baseline and 1 week after the IVR was 17.8 ± 12.5 for the treated eyes and −6.63 ± 10.6 for the untreated eyes (*P* = 0.005, paired *t* test). For the BRVO group, the mean baseline CMT was 568.0 ± 199.24 μm, and it significantly decreased to 391.0 ± 91.32 μm at 1 week after the IVR (*p* = 0.04).

### Significant decrease in dMBR in treated eye but not in untreated eye

The mean MBR of the treated eyes was 15.54 ± 3.74 at the baseline, and it decreased significantly to 12.64 ± 4.06 at 1 day after treatment and to 13.62 ± 3.49 at 1 week after treatment (*P* < 0.01, repeated ANOVA). However, there were no significant changes in the untreated eyes; 12.86 ± 2.43 at the baseline, 12.87 ± 2.50 at 1 day, and 13.07 ± 2.81 at 1 week after treatment (*P* > 0.05; repeated ANOVA).

The dMBR at 1 day after IVR was −16.4 ± 17.0% in the treated eyes and 2.31 ± 19.3% in the untreated eyes (*P* = 0.01, paired *t* test, Fig. 2b). The dMBR at 1 week after treatment was −12.0 ± 14.6% in the treated eyes and 4.50 ± 25.9% in the untreated eyes (*P* = 0.03, paired *t* test, Fig. 2c).

### Significant decrease in dMBR in treated eyes but not in untreated eye for DME

**Fig. 3:**
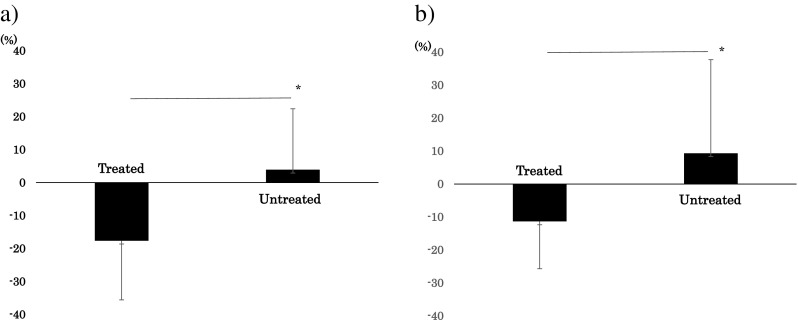
Comparison of the changes in the relative MBR after IVR in an eye with diabetic macular edema. **a** There is a significant difference in the relative MBR of the treated and untreated eyes at 1 day after IVR (*P* = 0.02, paired *t* test). **b** There is also a significant difference in the relative MBR of the treated and untreated eyes at 1 week after the IVR injection *(P* = 0.03, paired *t* test, **b**). *IVR*, intravitreal ranibizumab; *MBR*, mean blur rate

Because the number in the BRVO group were four, we examined for the DME group. The dMBR in the DME group (11 eyes) at 1 day after IVR was −17.6 ± 17.9% for the treated eyes and 3.91 ± 18.5% for the untreated eyes (*P* = 0.02, paired *t* test, Fig. 3a). The dMBR at 1 week after treatment was −11.4 ± 14.3% for treated eyes and 9.32 ± 28.4% for untreated eyes (*P* = 0.02, paired *t* test, Fig. 3b).

### Case presentation

**Fig. 4:**
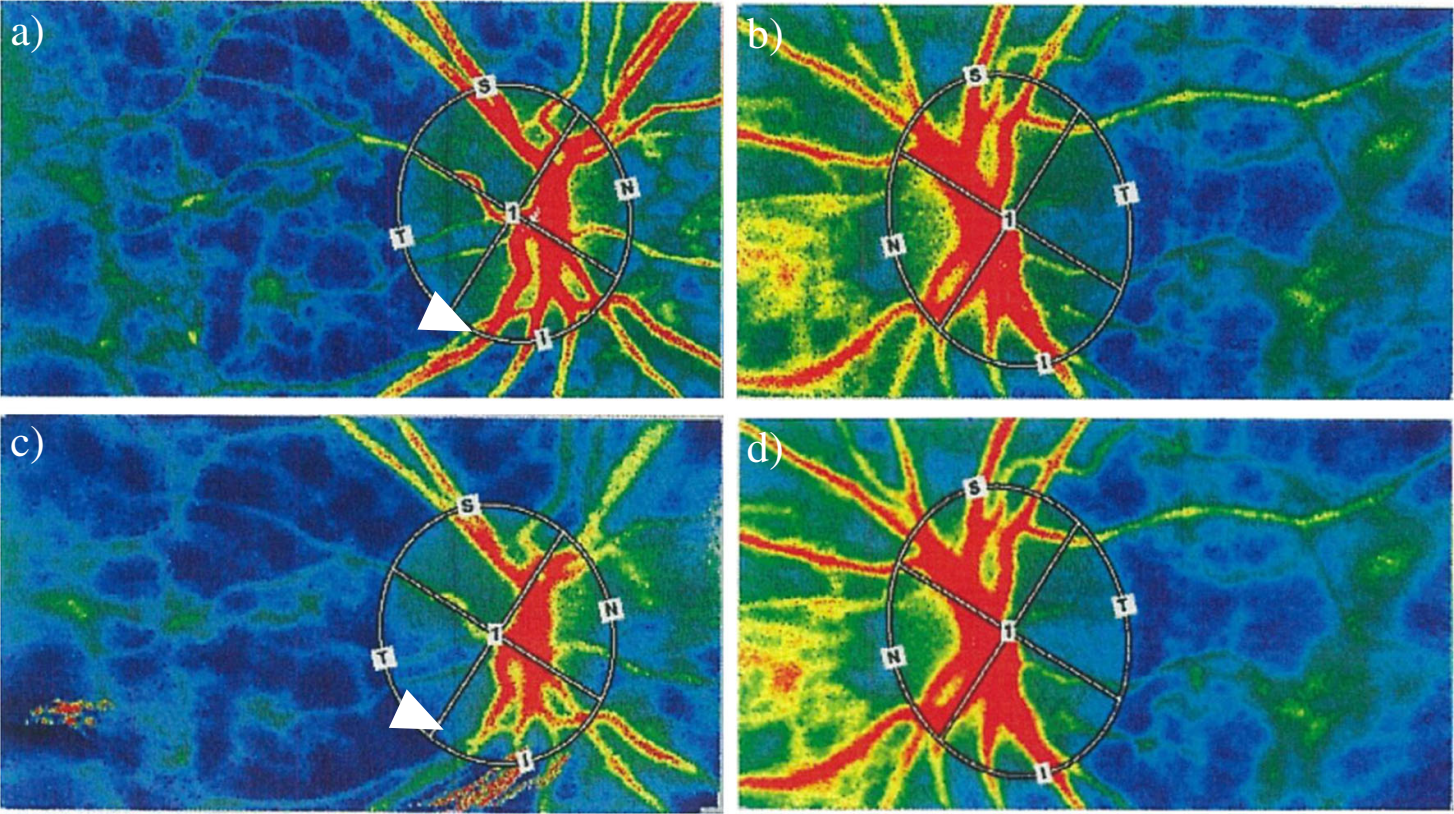
Representative case with diabetic macular edema. Images from a 63-year-old woman with diabetic macular edema in her right eye. Color LSFG map of the right eye (**a**, **c**) and left eye (**b**, **d**) are shown. Before the IVR in the right eye, the MBR was 17.8 for her right eye (treated eye, **a**) and 17.8 for the left eye (untreated eye, **b**). One day after treatment, the MBR decreased to 13.0 (**c**) in the injected eye and increased slightly to 18.9 in the untreated eye (**d**). *White arrow* indicates narrowness of same vessel before and after treatment. *LSFG*, laser speckle flowgraphy; *MBR*, mean blur rate

A representative DME case is shown in Fig. 4. The patient was a 63-year-old woman with DME in her right eye only, and she received an IVR injection. The decimal BCVA before treatment was 0.6 and the MBR was 17.8 for right eye. For the non-treated eye, the decimal BCVA before treatment was 0.7 and MBR value was 17.8. One day after treatment, the decimal BCVA improved to 0.7, but the MBR value decreased to 13.0 for the treated eye (Fig. 4a, c). For the untreated eye, the decimal BCVA was 0.6 and MBR was 18.9 1 day after treatment (Fig. 4b, d). Because the basic status of each eye is not necessarily the same, we used dMBR as a rate of change to compare the MBR change during treatment. The dMBR was −27.0% for the treated eye and 6.18% for the untreated eye. For the treated eye, regression to warm color which represents MBR regression was seen, and no remarkable change was seen for the untreated eye. Thus, the blood circulation in the treated eye was decreased, but not in the untreated eye.

## Discussion

Intravitreal injections of anti-VEGF agents can resolve the macular edema, subretinal fluid, and neovascularization very rapidly [22, 23]. Previous studies using Doppler imaging on eyes with AMD and DME showed that the retrobulbar circulation decreased after a bevacizumab injection [24, 25]. A LSFG study also found a decrease in the ocular circulation after an intravitreal anti-VEGF agent injection in the treated eye at different stages of diabetic retinopathy (DR) [26], and MBR was reported to be significantly correlated with the foveal thickness [21]. These findings indicate that anti-VEGF agents affect the ocular circulation associated with a reduction of the CMT. Anti-VEGF agents are effective not only for ME resolution but also on vascular contraction. This fact indicates that the vascular autoregulation works well for eyes with a resolution of the ME. There is a possibility that these changes are related to the ME regression and the integrity of the blood vessels. Although an intravitreal injection of anti-VEGF agents affected the blood flow strongly, it only affected the treated eyes and not the fellow untreated eyes. Other reports did not mention the circulation of the untreated eyes. Here, we found that dMBR decreased for treated eyes and slightly increased for untreated eyes. The reason for the increase in the untreated eye is not clear, but because this increase was not significant from comparison of raw MBR value, so we can ignore this change.

Anti-VEGF agents are also used for anti-cancer therapy. Some studies have reported that they may increase the coagulopathy complications [27, 28]. Systemic administration leads to higher levels because the usual dose is 10 mg/kg (Avastin Medical insert, Genentech, San Francisco, CA, USA) which is much higher than the 1.25 mg intravitreal injection. This indicates that systemic complications are less likely to occur after intraocular injections. After intravitreal triamcinolone acetonide injections, the systemic concentrations of corticosteroid were not altered, which suggests that agents that are given intravitreally generally do not enter the systemic circulation in effective concentrations [29]. However, the pharmacokinetics of drugs differ, and it is difficult to compare them. Anti-VEGF agents are also approved for DME, although it is well known that vascular infarction is a major compilation for diabetic patients, and painless myocardial infarction or micro-cerebral infarction is also higher in diabetic patients [30, 31]. There have been at least two studies on the systemic risks of intravitreal anti-VEGF agents for a high-risk group and a diabetes group [32, 33]. Though we found no obvious systemic changes including any thromboembolism event during the observation, even in the DME group, we need to pay special attention to diabetic patients.

At present, many different anti-VEGF agents are available. Their properties are somewhat similar, but the molecular conformation and target receptors are different. The different properties can affect their kinetics in the retina [34, 35] and clinical efficacies [36, 37]. Some reports on the systemic concentration of VEGF are different for bevacizumab, ranibizumab, and aflibercept (Eylea®, Bayer, Leverkusen, Germany), and the systemic VEGF concentration is not different only after ranibizumab treatment [38–40]. These results indicate that the clinical effects and systemic complications may be different between anti-VEGF agents. Previous experimental and clinical studies have also reported pharmacokinetics of ranibizumab, and imply that ranibizumab has little influence on systemic VEGF concentration [41–43]. It is not clear whether ranibizumab does not penetrate from vitreous to blood circulation or does not have a effect though it circulates throughout the body, but there is a possibility that ranibizumab is a safe agent among anti-VEGF agents. Though the systemic circulation of anti-VEGF agents after intravitreal injections is not high, it is better to use a safer drug to avoid systemic complications.

There are limitations in our study including the small sample size. Although we did not detect any change in the fellow eye, this does not necessarily mean the IVR had not flowed into the systemic circulation, because the concentration may not be high enough to affect the ocular circulation and cause systemic side-effects. In addition, it has been reported that unilateral intravitreal aflibercept affected the fellow eyes without systemic complications [44–46].

A second limitation is that we did not evaluate the systemic VEGF concentration or coagulating fibrinogenolysis system. Third, we measured the MBR of only the ONH. Generally, LSFG can evaluate the ONH which represents the blood flow of the entire retina, individual blood vessels, and the macular circulation which represents the choroidal blood flow. For the evaluation of individual blood vessels, it is difficult to compare vessels between patients because each position or diameter is different. For the macular area,positioning or focus are also difficult because of the presence of edema. And its reproducibility is reported to be good on the ONH, but is not good on the vessels [21]. Thus, we used the ONH because of its accessibility and reproducibility. But it is also important to evaluate the choroidal circulation which we examined by another technique to evaluate the ocular circulation more accurately.

In conclusion, our results showed that an IVR injection affected the ocular circulation of only the treated eyes and did not affect the ocular circulation of the fellow eyes. There is a possibility that ranibizumab does not enter the systemic circulation in concentrations at a high enough level to affect the circulation of the contralateral eye. But the true mechanisms which cause systemic complications after anti-VEGF treatment are still not clear, so further investigation is needed.
